# mt‐Ty 5'tiRNA regulates skeletal muscle cell proliferation and differentiation

**DOI:** 10.1111/cpr.13416

**Published:** 2023-02-08

**Authors:** Jun Cao, Xin Wang, Vivek Advani, Yao Wei Lu, Andrea P. Malizia, Gurinder Bir Singh, Zhan‐Peng Huang, Jianming Liu, Chunbo Wang, Edilamar M. Oliveira, John D. Mably, Kaifu Chen, Da‐Zhi Wang

**Affiliations:** ^1^ Department of Cardiology, Boston Children's Hospital Harvard Medical School Boston Massachusetts USA; ^2^ Faculty of Environment and Life Beijing University of Technology Beijing P. R. China; ^3^ Departments of Internal Medicine, Molecular Pharmacology & Physiology, Center for Regenerative Medicine, USF Health Heart Institute, Morsani College of Medicine University of South Florida Tampa Florida USA; ^4^ Vascular Biology Program, Department of Surgery, Boston Children's Hospital Harvard Medical School Boston Massachusetts USA; ^5^ UNC McAllister Heart Institute University of North Carolina Chapel Hill North Carolina USA; ^6^ School of Physical Education and Sport University of Sao Paulo Sao Paulo Brazil; ^7^ Present address: Vertex pharmaceuticals Boston Massachusetts USA

## Abstract

In this study, we sought to determine the role of tRNA‐derived fragments in the regulation of gene expression during skeletal muscle cell proliferation and differentiation. We employed cell culture to examine the function of mt‐Ty 5’ tiRNAs. Northern blotting, RT‐PCR as well as RNA‐Seq, were performed to determine the effects of mt‐Ty 5’ tiRNA loss and gain on gene expression. Standard and transmission electron microscopy (TEM) were used to characterize cell and sub‐cellular structures. mt‐Ty 5’tiRNAs were found to be enriched in mouse skeletal muscle, showing increased levels in later developmental stages. Gapmer‐mediated inhibition of tiRNAs in skeletal muscle C2C12 myoblasts resulted in decreased cell proliferation and myogenic differentiation; consistent with this observation, RNA‐Seq, transcriptome analyses, and RT‐PCR revealed that skeletal muscle cell differentiation and cell proliferation pathways were also downregulated. Conversely, overexpression of mt‐Ty 5’tiRNAs in C2C12 cells led to a reversal of these transcriptional trends. These data reveal that mt‐Ty 5’tiRNAs are enriched in skeletal muscle and play an important role in myoblast proliferation and differentiation. Our study also highlights the potential for the development of tiRNAs as novel therapeutic targets for muscle‐related diseases.

## Graphical abstract

tiRNAs are tRNA halves derived from cleavage of pre‐ or mature tRNAs. We identified and characterized a specific tiRNA group in skeletal muscle (mt‐Ty 5’tiRNAs). We determined that inhibition of mt‐Ty 5’tiRNAs reduced cell proliferation and myogenic differentiation.

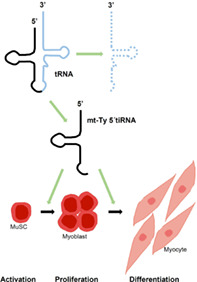

## 1 INTRODUCTION

Transfer RNAs (tRNAs) are essential components of the protein synthesis machinery. tRNA‐derived small RNAs (tsRNAs) are a recently identified category of small non‐coding RNAs (sncRNAs) that are generated by cleavage of pre‐ or mature tRNAs.^1^ Although the nomenclature of tsRNAs has not yet been standardized,^2^, ^3^, ^4^ they are mostly categorized into two groups: (1) tRNA halves (or tRNA‐derived, stress‐induced RNAs, referred to as tiRNAs) and (2) tRNA‐derived fragments (tRFs). tiRNAs are typically 30–50 nt long which is half the size of typical tRNAs.^1^ As indicated by their name, tiRNAs have been found to be induced by stresses, such as arsenites, heat shock, and ultraviolet irradiation.^5^ Angiogenin (ANG), a tRNA‐specific ribonuclease, is required for stress‐induced production of tiRNAs both in vitro and in vivo.^6^ ANG cleaves tRNAs in the anticodon region, and leaves 2′‐ 3′‐ cyclic phosphates at the 3′ ends and hydroxyl groups at 5′ ends of tRNAs^5^; these are the unique characteristics of tiRNAs that differentiate them from tRFs. tiRNAs have been found to be involved in the regulation of protein translation^3^, ^6^ and mRNA stability^7^; they also participate in multiple biological processes such as stem cell differentiation,^7^ cell proliferation,^8^ apoptosis^9^ and immune response.^10^ However, little is known about whether and how tiRNAs are involved in the regulation of skeletal muscle development.

Skeletal muscle development involves highly coordinated and complex molecular mechanisms and pathways, including myogenic regulator activation,^11^ signalling transduction,^12^ cell cycle arrest,^13^ mitochondrial biogenesis,^14^ and stress response.^15^ Skeletal muscle differentiation is tightly accompanied by stress processes including reactive oxygen species production, DNA damage response, mitochondrial fission, and autophagic and mitophagic flux.^16^ The continued identification and characterization of novel regulators of skeletal muscle development and function will be instrumental for the design of new therapeutic approaches for the treatment of muscle diseases.

In this study, our goal was to investigate the role of sncRNAs in skeletal muscle. We initially observed an enrichment of 5'tiRNAs in 2‐month‐old (2mo) mouse skeletal muscle, from which mt‐Ty 5'tiRNAs were identified to be the most abundant. Therefore, we inhibited or overexpressed mt‐Ty 5'tiRNAs in the mouse skeletal muscle C2C12 myoblast cell line to assess their function. We determined that depletion of mt‐Ty 5'tiRNAs led to reduced cell proliferation and differentiation, induced apoptosis and mitochondrial fragmentation. Correspondingly, the expression of genes involved in muscle cell growth and development was altered. These results demonstrated that precise regulation of the levels of mt‐Ty 5'tiRNAs are required for muscle cell proliferation and differentiation.

## 2 MATERIALS AND METHODS

### 2.1 Cell culture

C2C12 myoblast cells (CRL‐1772) were grown and maintained at 50% confluency in Dulbecco's modified Eagle's medium, supplemented with 10% fetal bovine serum and 100 units/ml penicillin and streptomycin. For transfection, cells were seeded at ∼60%–70% confluency; after 4 h, they were transfected with gapmers or tiRNA mimics using RNAiMAX (Thermofisher Scientific 13,778,150). The concentration for the treatment of gapmers and tiRNA mimics was 50 nM and 500 μM, respectively. Subsequent experiments including RNA‐Seq and immunofluorescent staining were conducted after 24 h of transfection unless otherwise indicated. For experiments conducted using differentiating C2C12 cells, the cells were switched to a medium containing 2% horse serum to induce differentiation on the day that the cells reached 100% confluency; samples were collected on the indicated dates during differentiation. Myogenesis was monitored by staining cells with myogenic markers. Cells containing two or more nuclei were recorded as myotubes.

### 2.2 RNA preparation and analysis

RNAs were extracted from tissue or cells using TRIzol (Thermofisher Scientific 15,596,018) following the manufacturer's protocol. For RNA‐Seq, the amount and quality of RNA were determined by Agilent TapeStation (Agilent). Only the RNAs with DV200 value >70 and RIN >8 were used for RNA‐Seq. For Northern blot and RT‐PCR, the amount and quality of RNA were determined by Nanodrop and only the RNAs with 260/280 > 1.9 were used for those experiments.

### 2.3 Plasmid DNA deep sequencing

Total RNAs from 2mo BL/6 mice were extracted as described above and analysed on a 15% denaturing polyacrylamide gel (PAGE) gel. RNA bands were stained by ethidium bromide (EtBr), and the area around 20–100 nt was cut and eluted with 0.3 M NaCl. Eluted RNA was ligated to a Linker with RNA ligase, then purified and linked to 3′ adaptors. After another round of purification, reverse transcription was performed with the 3′ antisense primer and PCR was performed with both the 5′ and 3′ primers. Finally, PCR products were used to quickly ligate using the TOPO system (Thermofisher Scientific). After transformation in ultracompetent cells, colonies were selected, and mini‐preps were prepared for plasmid DNA deep sequencing.

### 2.4 Near‐infrared fluorescent northern blot and DIG northern blot

The mitochondrial tyrosine tRNA (mt‐Ty) tRNAs and mt‐Ty 5'tiRNAs in skeletal muscle of 2mo mice were determined by near‐infrared fluorescent northern blot.^17^ Briefly, 10–20 μg of total RNA from each sample was separated on 15% Urea‐PAGE gel and transferred to Hybond N+ membrane (GE). The membrane was crosslinked twice using a 254 nm UV crosslinker at 120 mJ/cm^2^, which was followed by incubation with ExpressHyb hybridization solution (Takara) for 30 min at 30°C. The membrane was then hybridized overnight at 30°C with IR‐dye conjugated probes (Table S1). To conjugate the probes with IR‐dye, 2.5 nmol oligos were combined with 50 nmol IRDye 680RD (Li‐Cor, catalogue number: 929‐50005) or 800CW DBCO (Li‐Cor, catalogue number: 929‐55000) in PBS with a reaction volume of 50 μl, then incubated at room temperature in the dark for 6 h. After incubation, 2 volumes of AMPure XP beads (Beckman Coulter, catalogue number: A63881) and 5.4 volumes of isopropanol were mixed with the reaction to purify the IR‐dye conjugated probes. The membrane was washed twice with 1X SSC‐0.1% SDS buffer at room temperature on the following day and scanned on an Amershan Typhoon scanner (GE health) to detect emission at 600 nm and 800 nm.

### 2.5 qPCR analysis

To determine changes in tiRNA levels, total RNAs were ran on a TBE‐Urea gel and 10–50 nt bands were excised for RNA isolation. RNAs were reverse transcribed and amplified using the NCode miRNA amplification system (Thermofisher Scientific). qRT‐PCR was performed to establish transcript levels of these and other downstream target genes discussed in the manuscript (primer source, IDT/Integrated DNA Technologies). Products were measured by absolute quantification and reported as a function of cycle threshold (*Ct*). mRNA expression was normalized to U6 expression, as reported in the text, thus obtaining relative expression (Δ*Ct*) and mean fold change values (ΔΔ*Ct*). Following cycling, to ensure specificity, a melting curve analysis was carried out to verify the amplification of PCR products. The presence of one peak in the melting curve was employed as a requirement to ensure the absence of secondary products. Fold differences were calculated over control for each exposed group using normalized *Ct* values.

### 2.6 RNA‐Seq analysis

Non‐strand‐specific poly‐A selected libraries were generated using the TruSeq RNA Library Preparation Kit (Illumina), followed by paired‐end sequencing (PE150bp, GENEWIZ). The mouse genome version mm10 and the associated reference gene version were downloaded from the 10Xgenomics site (https://cf.10xgenomics.com/supp/cell-exp/refdata-gex-mm10-2020-A.tar.gz). We trimmed adapter sequences and low‐quality sequences from the RNA‐Seq data using Trim Galore v0.6.6 (Martin 2011) with default parameters. The high‐quality reads were mapped to the mouse genome version mm10 using TopHat v2.1.1 (Kim et al., 2013), which was guided by gene models. Successfully mapped reads were sorted, and unique mapped reads were retained by SAMtools v1. 5. Read counts for each gene were quantified by Htseq‐count v0.11.2 (Anders et al., 2015) with union gene region option. Afterwards, we used edgeR to normalize read counts and detect differentially expressed genes (DEGs) between mt‐Ty 5'tiRNAs gapmer treated samples and control samples, as well as between 5' tiRNA overexpression samples and control samples. We defined DEGs as those with a fold change greater than 1.5 and an FDR value smaller than 0.05. Gene ontology (GO) analysis was performed to check the functional enrichments of DEGs by the R package clusterProfiler v3.18.1 (Wu et al., 2021). Gene Set Enrichment Analysis was performed using GSEA Java software. Heatmaps and volcano plots were ge 0063nerated by pheatmap (Kolde, 2012) and ggplots, respectively.

### 2.7 Transmission electron microscopy

To obtain ultrastructural analysis of cells in ultrathin sections, C2C12 myoblasts were fixed for 24 h in a mixture of 1.25% formaldehyde, 2.5% glutaraldehyde, and 0.03% picric acid in 0.1 M sodium cacodylate buffer, pH 7.4. The fixed cells were washed with 0.1 M sodium cacodylate buffer and post‐fixed with 1% osmium tetroxide/1.5% potassium ferrocyanide (in H_2_O) for 2 h. Samples were then washed in a maleate buffer and post fixed in 1% uranyl acetate in maleate buffer for 1 h. Following the wash, the cells were rinsed in ddH_2_O and dehydrated through a series of ethanol treatments (50%, 70%, 95%, 100% × 2 times) for 15 min per solution. Dehydrated samples were processed through propylene oxide and centrifuged into pellets. They were then infiltrated with epon mixed 1:1 with propylene oxide overnight at 4°C. Samples were allowed to polymerize in epon resin at 60°C in an oven for 48 h. They were then sectioned into 80 nm thin sections using a Leica UC7 Ultramicrotome and imaged on a Tecnai™ G^2^ Spirit BioTWIN transmission electron microscope.

### 2.8 Immunofluorescent staining of cells

Cells were plated into Millicell EZ SLIDE 8‐well glass (Millipore PEZGS0816) at a density of 2 ×10^4^ cells/well and transfected with gapmers (Table S1) at 50 nM for 24 h. Cells were fixed in 4% PFA and blocked with blocking buffer (PBS/5% normal serum/0.3% Triton™ X‐100) at room temperature for 60 min. Cells on slides were incubated with primary antibody at the dilutions indicated in the next section in antibody dilution buffer (PBS/1% BSA/0.3% Triton™ X‐100) at 4°C overnight. After primary antibody incubation, the cells were incubated with fluorochrome‐conjugated secondary antibodies according to the manufacturer's instructions at room temperature for 1 h in the dark. The slides were then washed with PBS three times for 10 min each, then mounted with Prolong® Gold Antifade Reagent with DAPI (Vector Laboratories, H‐1200‐10).

### 2.9 Microscopy imaging and analysis

Bright‐field images in Figures 2A and 3D were captured for live cells using Keyence BZ‐X710 all‐in‐one fluorescence microscope (Keyence Corporation, Osaka, Japan). For images in Figures 2C, D, 3A, E, F, 5A, B, cells were fixed and stained as described above, then imaged using the same Keyence instrument noted above. All other samples for fluorescent imaging were prepared using the fixation and staining procedures described above and imaged using an Olympus Fluoview FV3000 confocal laser scanning microscope (Olympus Corporation, Tokyo, Japan). Fluorescence intensity was measured using ImageJ and cell numbers were measured manually. For quantification, 9–10 images were taken for each sample, and three independent experiments (*n* = 3) were performed. The antibodies used for these experiments were: anti Ki‐67 (1:500, Cell Signalling, mAb #9449), phospho‐Histone H3 (1:500, Cell Signalling, mAb # 9706), Tomm20 (1:500, abcam, ab56783), MYH (1:200, Santa Cruz, sc‐376,157), MF20 (1:200, ThermoFisher, sc‐376,157).

### 2.10 Mitochondrial morphology analysis

Analysis of mitochondrial network connectivity by immunofluorescent staining in individual cells was performed using the MiNA plugin in ImageJ.^18^, ^19^ 2D microscopy images were pre‐processed using the ‘tophat filtering with a kernel convolution’ included in the MiNA plugin of the ImageJ package to enhance sharpness and reduced background noises of the images and then binarized to allow running the remaining features of the MiNa script. The measurement of individual mitochondria (mt), networks, volumes and branch length was determined using the available features of the MiNA module.

### 2.11 Quantification and statistical analysis

Quantification and statistical analysis for each experiment are detailed within each section of the figure legends. Graphpad Prism 6 Software was used to plot the graphs for all statistical analysis. Statistical significance was calculated using a *t*‐test to compare two different groups. Significance is defined as **p < 0.01 and ****p < 0.0001. For quantification of immunofluorescent experiments, *n* = 3 samples from three independent experiments. TEM images of mt were generated for *n* = 3 samples from three independent experiments. For quantification of surface area of mt and mitochondrial density, *n* = 128 mt for control gapmer treatment, *n* = 207 mt for mt‐Ty gapmer treatment.

## 3 RESULTS

### 3.1 Mt‐Ty 5'tiRNAs are enriched in skeletal muscle

**FIGURE 1:**
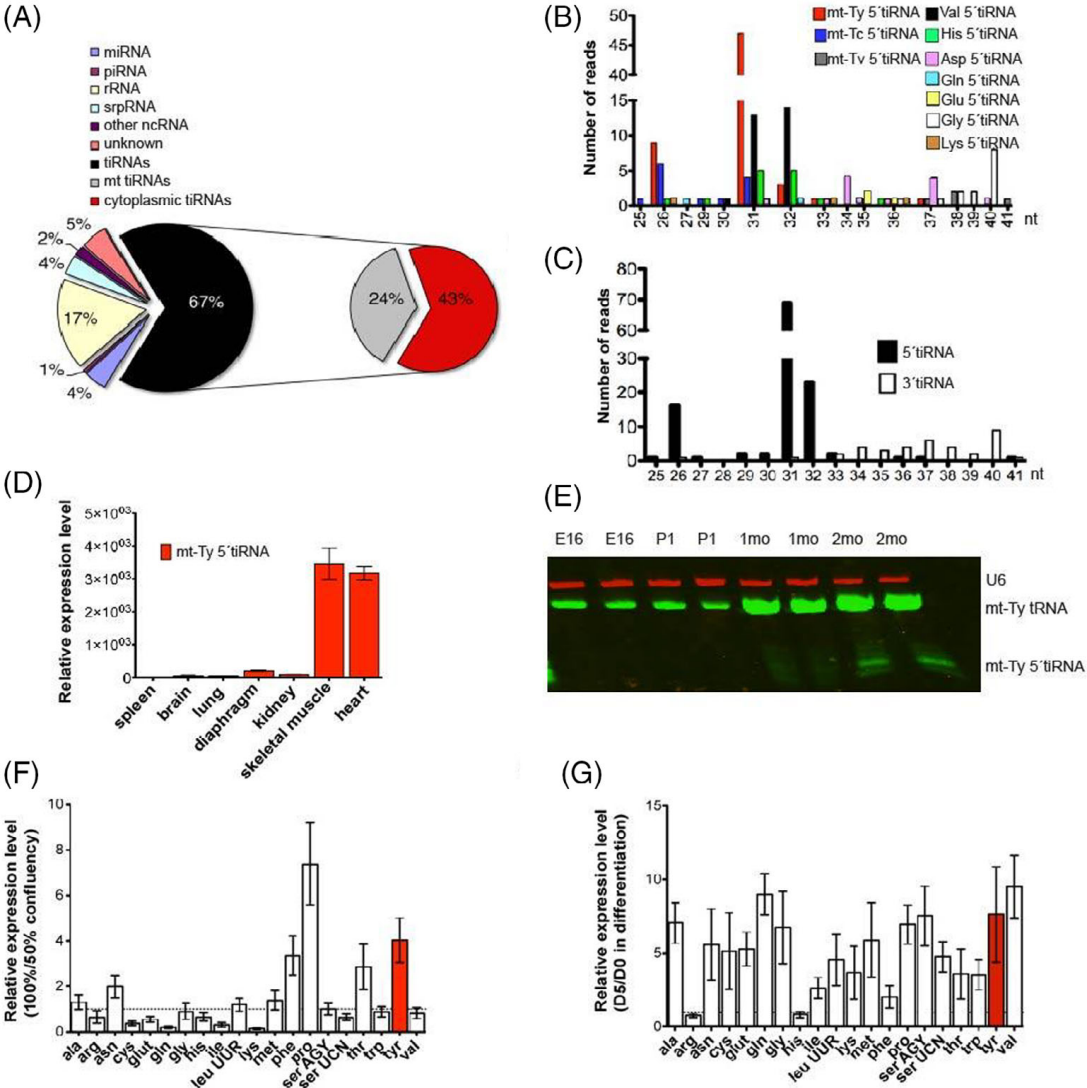
Mt‐Ty 5'tiRNAs are enriched in skeletal muscle during development. (A) Quantification of the number of 25–45 nt sncRNAs determined by deep sequencing of skeletal muscle from 2mo mice. miRNA, microRNA; piRNA, piwi RNA; rRNA, ribosomal RNA; signal recognition particle RNA (srpRNA), signal recognition particle RNA; tiRNA, tRNA‐derived, stress‐induced RNAs. (B) Number of reads of each 5'tiRNAs relative to length, as determined by deep sequencing. (C) Total quantification of 5'tiRNAs and 3'tiRNAs relative to the length of the fragment. (D) Quantification of mt‐Ty 5'tiRNAs in spleen, brain, lung, diaphragm, kidney, skeletal muscle, and heart of 2mo mice. (E) Identification of mt‐Ty tRNAs and mt‐Ty 5'tiRNAs of hind limb skeletal muscle from E16, P1, 1mo, and 2mo mice by near‐infrared fluorescent northern blot. (F) Quantification of mitochondrial tiRNAs in confluent (100%) C2C12 in comparison with proliferating cells (50%). U6 expression was used as a control (ΔΔ*Ct* = 1). Total RNA was isolated prior to the formation of myotubes. Experiments were performed in duplicate and are presented as average ± standard deviation. (G) Quantification of mitochondrial tiRNA in fully differentiated C2C12, using differentiation media for 5 days (D5), in comparison with proliferating cells (D0). U6 expression was used as a control (ΔΔ*Ct* = 1). Experiments were performed in duplicate and are presented as average ± standard deviation. The red bar in (F) and (G) indicates the expression level of Mt‐Ty 5'tiRNAs.

In the past, we have identified and investigated the expression and function of microRNAs (miRNAs) in skeletal muscle.^20^, ^21^ To identify additional sncRNAs in muscle, we harvested hind limb skeletal muscle tissue from 2mo BL/6 mice, extracted total RNA, and analysed the RNA using 15% denaturing PAGE. The area above the miRNAs (~ 21–23 nucleotide), and area corresponding to RNAs smaller than 45 nt, were excised from the gel and the RNA was eluted for cloning and sequencing, as described in the Materials and Methods. DNA‐sequencing of the cloned library and analysis revealed a list of sncRNAs, including mostly tiRNAs and ribosomal RNAs (rRNAs) which comprise 67% and 17%, respectively of the total sncRNA population, respectively (Figure 1A). In eukaryotic cells, the nucleus and mt both encode tRNA genes that contribute to the production of cytoplasmic tRNAs and mitochondrial tRNAs (mt‐tRNAs).^1^ The detected tiRNAs are derived from both mitochondrial and cytoplasmic tRNAs, which occupy 24% and 43% of sncRNAs, respectively (Figure 1A). The most highly enriched tiRNAs in skeletal muscle include Mitochondrial tyrosine (mt‐Ty), mitochondrial cysteine (mt‐Tc), mitochondrial valine (mt‐Tv), and cytoplasmic Val/His/Asp/Gln/Glu/Gly/Lys 5'tiRNAs (Figure 1B). The mt‐Ty 5'tiRNA, mt‐Tc 5'tiRNA, and Val 5'tiRNA are among the most abundant tiRNAs in skeletal muscle tissue (Figure 1B). Notably, 5'tiRNAs with lengths of ~26 and 31/32 nt are more abundant than tsRNAs of other lengths (Figures 1B, C), suggesting that the predicted cleavage site targeted by ANG is located in the anticodon loop, as previously reported.^5^ Moreover, 5'tiRNA and 3'tiRNA expression levels are not equally distributed; 5'tiRNAs are more abundant than 3'tiRNAs (Figure 1C), which is consistent with other published studies.^22^, ^23^

In the study by Dhahbi et al.,^22^ mt‐Ty 5'tiRNA was demonstrated to be the most abundantly expressed tiRNA in skeletal muscle; therefore, we decided to make this the focus of our study. We examined the tissue distribution of mt‐Ty 5'tiRNA expression in adult mice. We extracted RNAs from multiple tissues from 2mo mice, including the spleen, brain, lung, diaphragm, kidney, skeletal muscle, and heart. TaqMan‐based qPCR assays revealed that the mt‐Ty 5'tiRNA was primarily expressed in skeletal muscle and heart but barely detectable in other tissues (Figure 1D), suggesting a tissue‐specific expression pattern.

Next, we performed near‐infrared fluorescent northern blot^17^ using probes targeting the mt‐Ty 5'tiRNA to examine its expression from hind limb muscle tissues of mice at embryonic day 16 (E16), postnatal day 1 (P1), 1 mo, and 2 mo. mt‐Ty 5'tiRNA is detectable only in the samples from 1mo and 2mo mouse skeletal muscle with much higher expression found in skeletal muscle of 2mo mice (Figure 1E). (There is a slight shift of the bands from left to right as we allowed the gel bands to move further down to better resolve the different fragment sizes.) Notably, a comparable expression level of intact, mature mt‐Ty tRNA was found in skeletal muscle during all the time points from E16 to 2mo (Figure 1E). These data suggest that the expression levels of mt‐Ty 5'tiRNA and mt‐Ty tRNA are independently regulated, consistent with that of prior reports.^5^, ^7^, ^8^, ^24^

Using C2C12 mouse skeletal muscle myoblasts we examined the expression of mt‐Ty 5'tiRNA and other 5'tiRNAs during proliferation and differentiation. Cells were seeded at 50% confluency; the 5'tiRNA expression level was determined by qPCR using RNA isolated from a fraction of the cells collected during the proliferative state. The remainder of the cells (not used for the initial qPCR assay) were grown to confluency over the next 2–3 days; 5'tiRNA expression levels at 100% confluency were determined by qPCR as for the earlier samples. We found that the mt‐Ty 5'tiRNA expression levels were substantially higher in C2C12 myoblasts at 100% confluency versus 50% confluency (Figure 1F). Subsequently, we differentiated C2C12 myoblasts into myotubes and compared 5'tiRNA expression levels in 100% confluent C2C12 myoblasts versus in myotubes. For this experiment, the C2C12 myoblasts were allowed to reach 100% confluency, then the growth media was changed to differentiation media (which contains 5% horse serum), and the C2C12 cells were allowed to differentiate into myotubes. We defined the day we changed media as differentiation day 0 (D0). Upon switching to differentiating medium, increased mt‐Ty 5'tiRNA levels were detected in myotubes by differentiation day 5 (D5) (Figure 1G). These data are consistent with the results demonstrating higher mt‐Ty 5'tiRNA expression in skeletal muscle from 2mo mice.

### 3.2 mt‐Ty 5'tiRNAs regulate myoblast cell proliferation and differentiation

**FIGURE 2:**
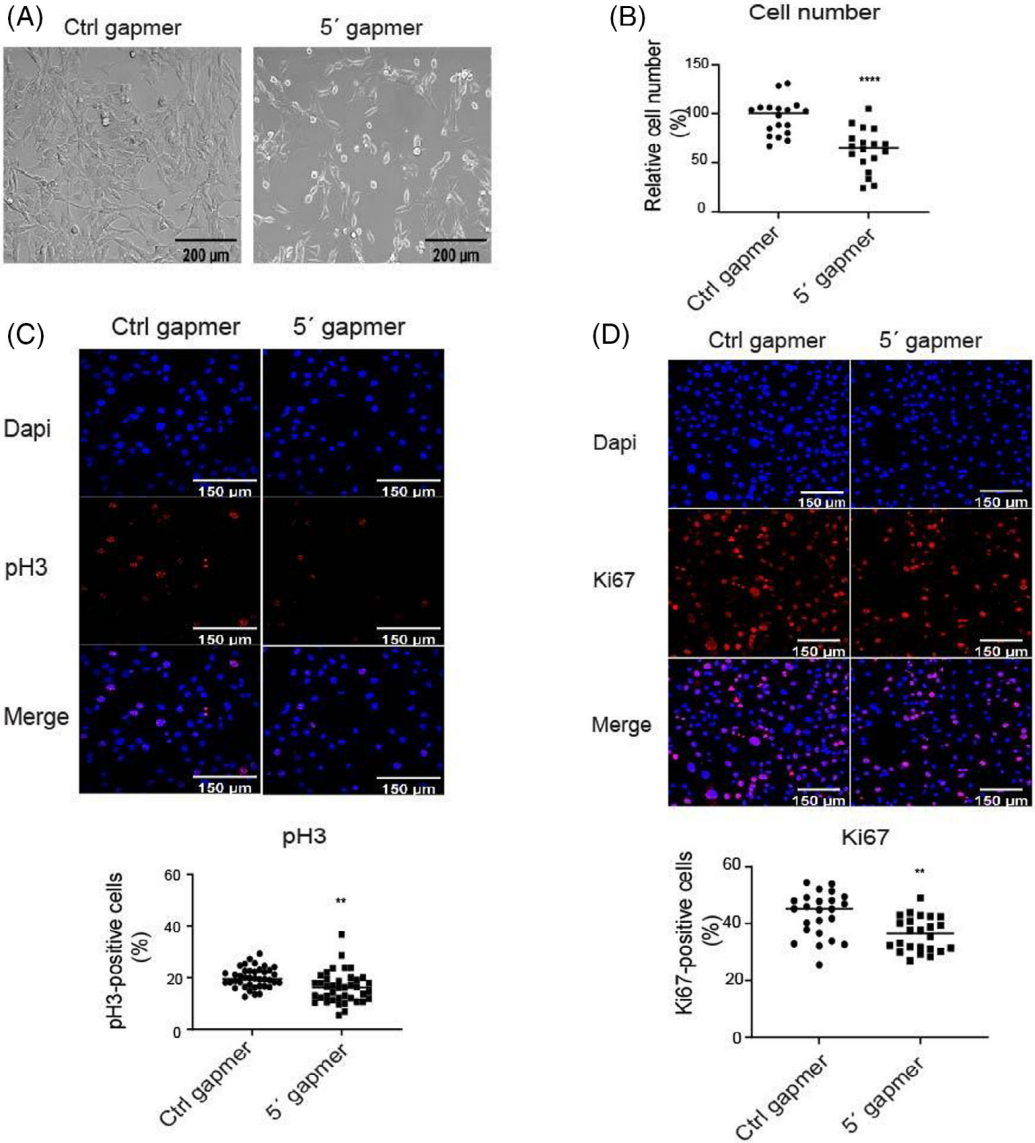
*Depletion of mt‐Ty 5'tiRNAs inhibited C2C12 proliferation*. (A) Representative bright field images of proliferating C2C12 myoblasts treated with Ctrl or 5'gapmer for 24 h. Scale bar =200 μm. (B) Relative cell number per field of 5'gapmer treated C2C12 myoblasts in comparison with Ctrl gapmer treated myoblasts (*n* = 3). *p* < 0.0001. (C) PH3 positive cells upon Ctrl versus 5′ gapmer treatment. Representative immunostaining image of cell proliferation marker pH 3 levels in C2C12 myoblasts treated with Ctrl or 5′ gapmer for 24 h (upper panel). Scale bar =150 μm. Quantification of the percentage of pH 3‐positive cells using ImageJ from the immunostaining images of pH 3 level in Ctrl versus 5'gapmer treated C2C12 myoblasts (*n* = 3) (lower panel). *p* = 0.0059. (D) Ki67 positive cells upon Ctrl versus 5′ gapmer treatment. Representative immunostaining images of cell proliferation marker Ki67 levels in C2C12 myoblasts treated with Ctrl or 5′ gapmer for 24 h (upper panel). Scale bar =150 μm. Quantification of the percentage of Ki67‐positive cells using ImageJ from the immunostaining images of Ki67 level in Ctrl versus 5'gapmer treated C2C12 myoblasts (*n* = 3) (lower panel). *p* = 0.0012.

To investigate the function of mt‐Ty 5'tiRNA in skeletal muscle, we designed an mt‐Ty gapmer whose sequence is complementary to mt‐Ty 5'tiRNA (Table S1), and transfected it into C2C12 myoblasts and examined the biological phenotypes. We seeded the C2C12 cells at 50%–60% confluency in the morning, and transfected mt‐Ty gapmers 6 h after seeding. We examined cell number and morphology the next day, 24 h after seeding. At this time point, the cells had typically reached 70% confluency (Figure 2A left panel), which is 1–2 days before cells reach 100% confluency. Upon treatment, Ctrl gapmer treated cells displayed a radial branching morphology and elongated appearance, whereas mt‐Ty gapmer treated cells were irregular in shape, with some elongated cells and others with a more rounded appearance (Figure 2A, right panel). We calculated the cell number by staining the cells with Dapi and counting with ImageJ software. At this stage, the cells were at 70% confluency or slightly less, and the nuclei had not yet started to fuse; therefore, the cells were easily resolved using this method (Figure 2C, D). We found that cell number was reduced by 34% upon mt‐Ty gapmer treatment (Figure 2B); To investigate the reason for the reduced cell numbers, we first examined the effect of mt‐Ty 5'tiRNAs on cell proliferation. We stained Ctrl and mt‐Ty 5′ gapmer treated cells with the phospho‐histone 3 (pH 3) and performed immunofluorescent imaging. PH3 is a proliferation marker which labels cells in mitosis and late G2 stages.^25^ We treated C2C12 cells with Ctrl and mt‐Ty 5'gapmers in three independent experiments (*n* = 3) and obtained 20 images per sample per experiment for quantification. We counted cell numbers using ImageJ software and calculated the pH 3 positive percentage by determining the number of pH 3‐stained cells and dividing by the total number of Dapi‐stained cells. Representative images of pH 3 staining are shown in Figure 2C upper panel. Our results showed that the number of pH 3 positive cells decreased from 20% to 16% after mt‐Ty gapmer treatment (Figure 2C, lower panel). To confirm our findings, we stained the two groups of cells with another cell proliferation marker Ki67 and performed immunofluorescent imaging by confocal microscopy. Ki67 is a proliferation marker which stains cells in the G1, S, S2, and M phases of the cell cycle.^25^ The pH 3 and Ki67 markers are complementary approaches used to establish the cell proliferation status. We treated C2C12 cells with Ctrl and mt‐Ty 5′ gapmers in three independent experiments (*n* = 3) and obtained 20 images per sample per experiment for quantification. We counted cell numbers with ImageJ software and calculated the Ki67 positive percentage by determining the number of Ki67‐stained cells and dividing by the total number of Dapi‐stained cells. Representative images of Ki67 staining are shown in Figure 2D upper panel. We found that Ki67‐positive cells dropped from 43% in Ctrl gapmer treated cells to 36% in mt‐Ty 5′ gapmer treated cells (Figure 2D, lower panel). These findings indicate that gamer‐mediated inhibition of mt‐Ty 5'tiRNA reduces myoblast proliferation.

### 3.3 Depletion of mt‐Ty 5'tiRNAs induced cell apoptosis and inhibited cell differentiation

**FIGURE 3:**
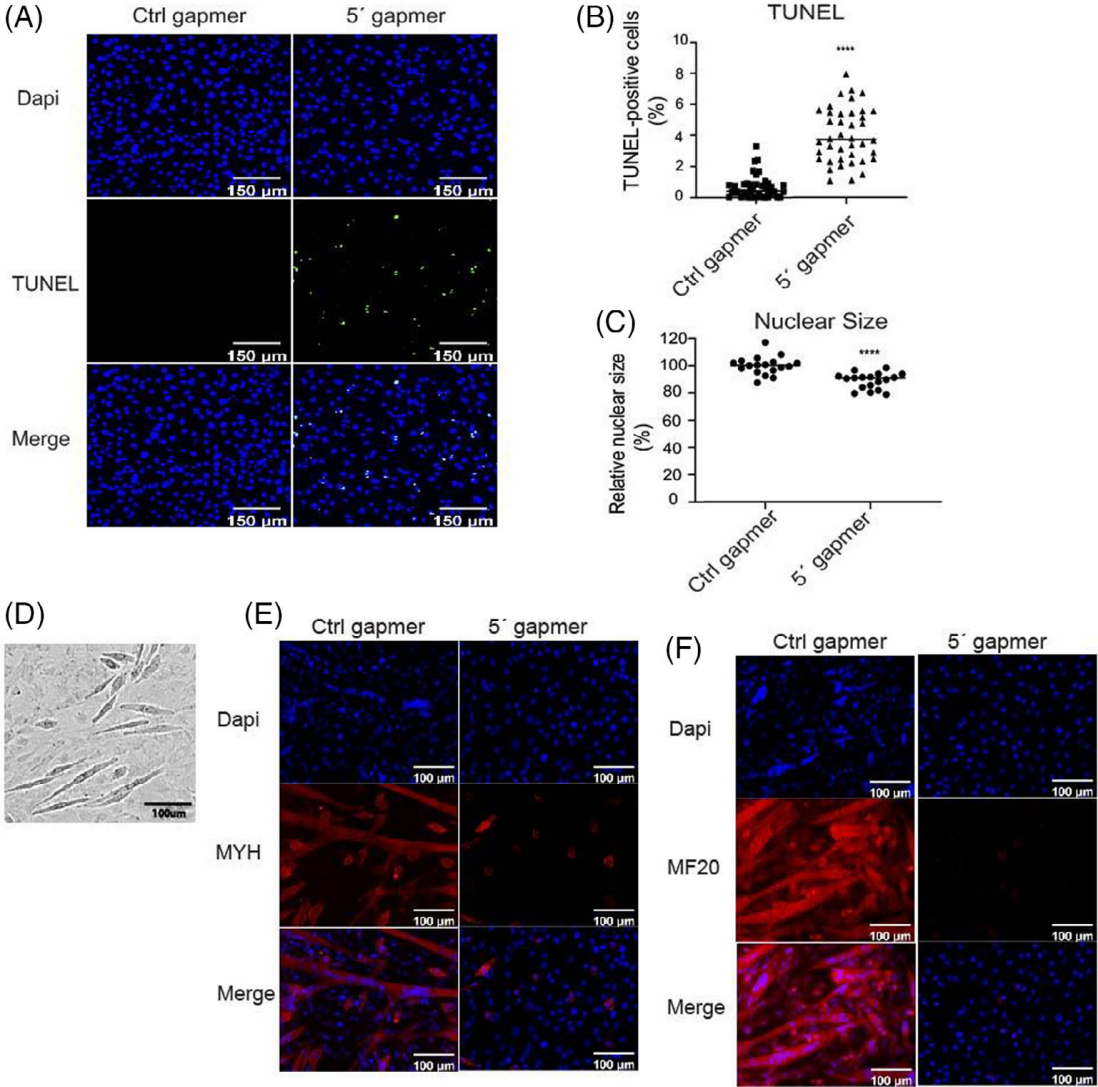
*Depletion of mt‐Ty 5'tiRNAs induced cell apoptosis and inhibited C2C12 differentiation*. (A) Representative images of TUNEL staining of C2C12 myoblasts treated with Ctrl or 5′ gapmer for 24 h. Scale bar =150 μm. (B) Quantification of the percentage of TUNEL‐positive cells using ImageJ from the immunostaining images of TUNEL level in Ctrl versus 5'gapmer treated C2C12 myoblasts (*n* = 3). *p* < 0.0001. (C) Relative nuclear size per field of 5'gapmer treated C2C12 myoblasts in comparison with Ctrl gapmer treated cells (*n* = 3). *p* < 0.0001. (D) Representative bright field images of C2C12 myotubes on D3 treated with Ctrl gapmers. Scale bar =100 μm. (E) Representative immunostaining image of late myogenesis marker MYH level in Ctrl gapmer or 5′ gapmer treated C2C12 myotubes on D3. Scale bar =100 μm. (F) Representative immunostaining image of marker MF20 in Ctrl gapmer or 5′ gapmer treated C2C12 myotubes on D3. Scale bar =100 μm.

Next, we examined the effects of gapmer treatment on cell apoptosis by TUNEL assay. To perform the TUNEL assay, cells were transfected with Ctrl or mt‐Ty 5'tiRNA gapmers 6 h after seeding. These cells were then fixed and permeabilized 18 h post‐transfection. Samples were incubated with TdT reaction buffer and the Click‐iT Plus TUNEL assay was performed according to the manufacturer's instructions. Positive controls were generated using DNAse I treatment. We imaged cells using confocal microscopy and counted TUNEL‐positive cells (stained with Alexa Fluor™ 488 picolyl azide dye) in each sample using ImageJ software. TUNEL positive cell percentage was determined by dividing the number of Alexa Fluor™ 488 stained cells by the total number of cells stained by Dapi. We found that 4% of the mt‐Ty 5′ gapmer treated cells were TUNEL positive, while no TUNEL‐positive cells were detected in the Ctrl gapmer treated cells (Figure 3A, B). Nuclear shrinkage is a typical feature of apoptosis^26^; therefore, we measured nuclear size and found that nuclear size was significantly decreased in mt‐Ty 5′ gapmer treated cells (Figure 3C). Therefore, our findings suggested that mt‐Ty 5′ gapmer treatment induced apoptosis.

Both apoptosis and proliferation are tightly associated with muscle differentiation and development^13^, ^27^, ^28^, ^29^; therefore, we further examined the effects of mt‐Ty gapmer treatment on C2C12 differentiation. We seeded C2C12 cells at 60%–70% confluency and transfected mt‐Ty gapmers 6 h later. We allowed the cells to reach 90%–100% confluency (48 h after seeding). We changed the growth media to differentiation media when the cells were fully confluent, and we defined this time point as D0. We maintained C2C12 myoblasts in differentiation media for 3 days to allow C2C12 myoblasts to differentiate into myotubes. Then we examined the cells by immunofluorescent staining using the differentiation markers MYH and MF20 on differentiation day 3 (D3). During differentiation, the C2C12 cells remained as a monolayer, while the cells started to fuse together on the plate. A representative bright field image is shown in Figure 3D. MYH and MF20 are antibodies that recognize different domains of myosin heavy chain; we used these antibodies to determine how mt‐Ty 5'tiRNAs affect muscle development since myosin heavy chain is enriched in mature myotubes.^30^ We found that myoblasts formed myotubes in Ctrl gapmer treated C2C12 cells where both MYH and MF20 were highly expressed (Figure 3E, F, left panels). In contrast, myoblast differentiation and myotube formation were blocked in mt‐Ty 5′ gapmer treated cells, correlating with the low levels of MYH and MF20 staining (Figure 3E, F, right panels). These results indicate that mt‐Ty 5'tiRNA is required for C2C12 myoblast differentiation.

### 3.4 mt‐Ty 5'tiRNAs regulate skeletal muscle differentiation pathways

**FIGURE 4:**
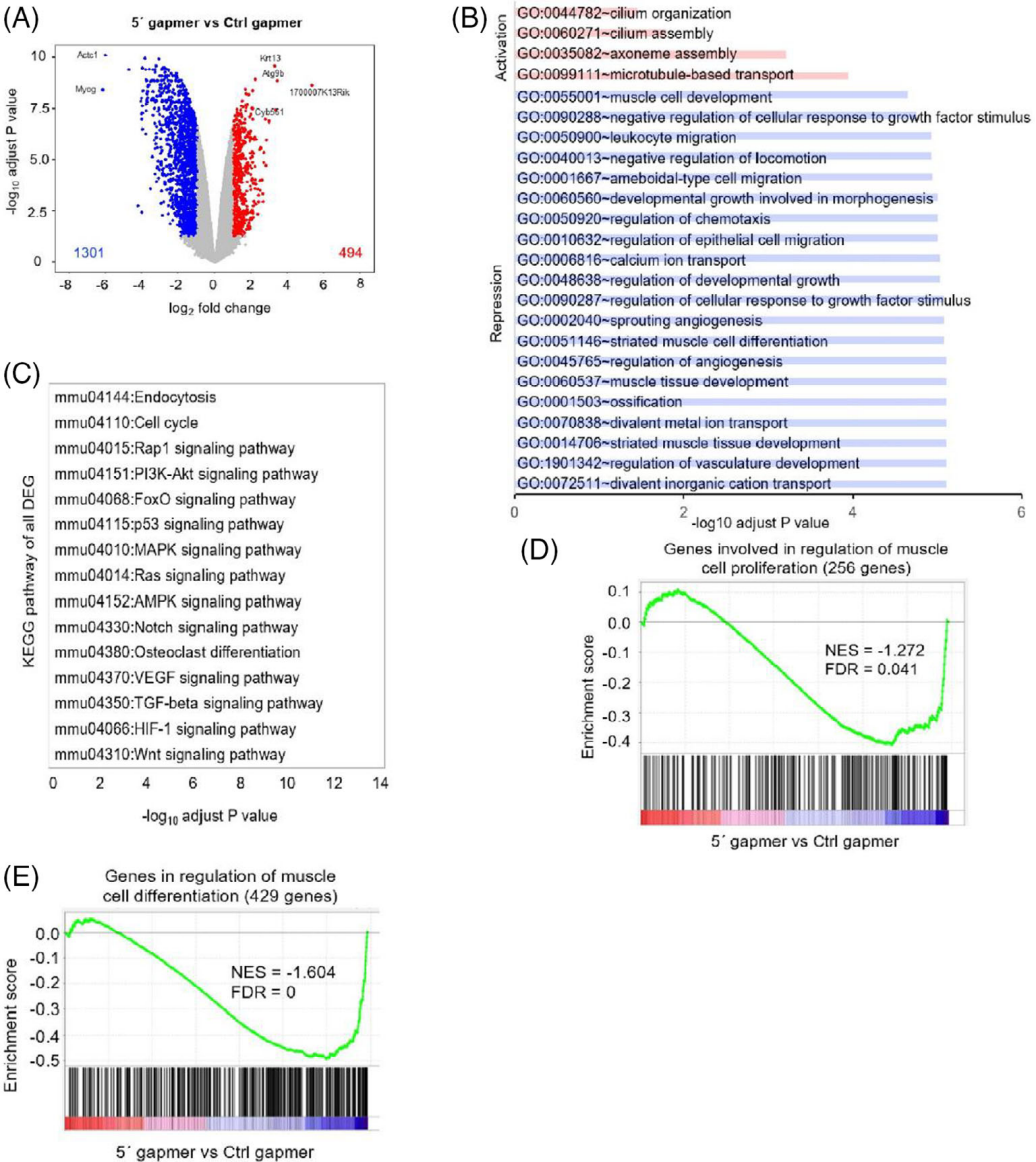
*Analysis of gene expression in cells with reduced levels of mt‐Ty 5' tiRNAs during proliferation*. (A) The volcano plot of gene expression changes in Ctrl gapmer versus 5′ gapmer treated C2C12 myoblasts while proliferating. Significantly reduced genes (Fold Change <−1.5, pAdj <0.1) are coloured blue; significantly activated genes (Fold Change >1.5, pAdj <0.1) are coloured red. (B) Gene ontology (GO) terms of reduced (blue) and activated (red) gene profile in C2C12 myoblasts with depletion of mt‐Ty 5' tiRNAs (pAdj). (C) KEGG pathways associated with differentially regulated genes in mt‐Ty 5′ gapmer treated cells. (D) GSEA analysis of genes differentially expressed in mt‐Ty 5′ gapmer treated C2C12 myoblasts reveals repression of cell proliferation genes. (E) GSEA analysis of genes differentially expressed in mt‐Ty 5′ gapmer treated C2C12 myoblasts reveals repression of muscle cell differentiation genes.

To gain a deeper understanding of the molecular function of mt‐Ty 5'tiRNA, we performed RNA‐Seq analysis in C2C12 myoblasts that were treated with Ctrl gapmer or mt‐Ty gapmer (Figure S1A). We obtained high‐quality data with a high mapping rate (~20 million reads per sample, >98% of high quality reads, and ~ 85% total mapping rate) (Figure S1B). Depletion of mt‐Ty 5'tiRNA resulted in dysregulation of 1795 genes; among them, 494 genes were upregulated and 1301 genes were downregulated compared to Ctrl gapmer treated cells (Figure 4A). Repressed genes were more abundant than activated genes when mt‐Ty 5'tiRNA was depleted, suggesting that mt‐Ty 5'tiRNA positively impacts gene expression. Among the most downregulated genes were Myog and Actc1, which encode the myogenic transcription factor *myogenin* and *alpha cardiac muscle 1 actin*, respectively (Figure 4A); their downregulation is consistent with our data showing that inhibition of mt‐Ty 5'tiRNA repressed myoblast differentiation. To validate our results, we performed qRT‐PCR and found that several proliferation and differentiation genes including Myog, Pax7, Atoh8, Mod1, Actn3, Ttn, and Igfbp5 were significantly reduced, consistent with the results of the RNA‐Seq (Figure S2).

GO‐enriched terms for upregulated genes include cilium organization and assembly, axoneme assembly and microtubule‐based transport, suggesting that loss of mt‐Ty 5'tiRNA may enhance cell migration (Figure 4B). In contrast, GO enriched terms for downregulated genes revealed that pathways related to ‘muscle tissue development’, ‘striated muscle tissue development’, and ‘muscle cell differentiation’ were among those with the most decreased levels (Figure 4B), supporting an important role for mt‐Ty 5'tiRNA in muscle gene expression and skeletal muscle differentiation. Interestingly, KEGG pathway analysis of all differentially expressed genes (DEG) in mt‐Ty 5'tiRNA gapmer treated cells revealed that many important pathways related to endocytosis and the cell cycle (such as the PI3K‐Akt signalling pathway, FoxO signalling pathway, and p53 signalling pathway) were affected (Figure 4C).

GSEA analysis showed that genes involved in muscle cell proliferation (256 genes) were repressed in cells treated with the mt‐Ty 5'tiRNA gapmer (Figure 4D, NES = −1.272, FDR = 0.041). In addition, this analysis revealed that genes involved in muscle cell differentiation (429 genes) are also markedly downregulated in mt‐Ty 5'tiRNA gapmer treated cells (Figure 4E, NES = −1.604, FDR = 0), supporting a key role for mt‐Ty 5'tiRNA in regulating the skeletal muscle cell proliferation and differentiation programs. Together, these data strongly support a role for mt‐Ty 5'tiRNAs in skeletal muscle cell proliferation and differentiation and are consistent with the finding that depletion of mt‐Ty 5'tiRNAs hinders these processes in C2C12 cells.

### 3.5 Depletion of mt‐Ty 5'tiRNAs induces mitochondrial fragmentation

**FIGURE 5:**
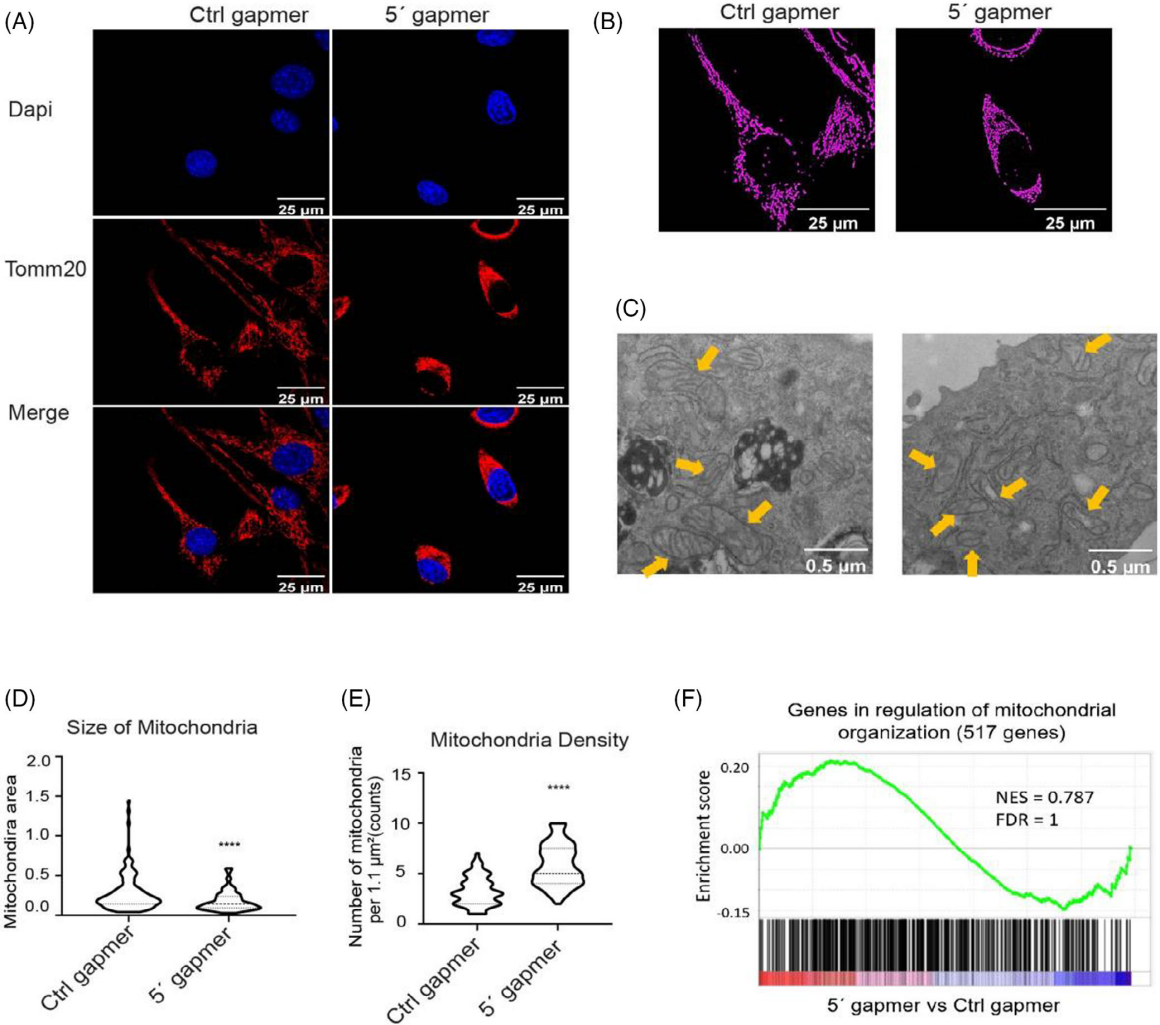
*Depletion of 5'tiRNAs induced mitochondria fragmentation*. (A) Representative immunostaining images of mitochondrial marker Tomm20 level in Ctrl or 5′ gapmer treated C2C12 myoblasts. Scale bar =25 μm. (B) Representative images depicting different mitochondrial network connectivity in Ctrl or 5′ gapmer treated C2C12 myoblasts processed by MiNA plugin in ImageJ. Scale bar =25 μm (C) Representative transmission electron microscopy (TEM) images of mitochondria in Ctrl or 5′ gapmer treated C2C12 myoblasts at a magnification of 6800×. Yellow arrows mark the mitochondria. Scale bar =0.5 μm. (D) Surface area of mitochondria in Ctrl versus 5'gapmer treated C2C12 myoblasts was calculated using ImageJ (*n* > 100 mitochondria were randomly selected and quantified in each group). Data represent means ± SD. Statistical significance was calculated using a *t*‐test to compare two different groups. ****p < 0.0001. (E) Mitochondria density was quantified as mitochondria number per 1.1 μm^2^ area in TEM images of Ctrl or 5′ gapmer treated C2C12 myoblasts (*n* = 36 square areas were randomly selected in each group for quantification). Data represent means ± SD. Statistical significance was calculated using a *t*‐test to compare two different groups. ****p < 0.0001. (F) GSEA analysis of genes differentially expressed in mt‐Ty 5′ gapmer treated C2C12 myoblasts reveals no significant changes in mitochondrial organization‐related genes.

Since mt‐Ty was initially annotated as mitochondrial tRNA, we characterized the role of mt‐Ty 5'tiRNA in this organelle. We stained proliferating C2C12 myoblasts with Tomm20, which stains the outer mitochondrial membrane, and imaged them using an Olympus FV3000RS inverted laser scanning confocal microscope with a 60X/1.3 silicone oil objective. The mitochondrial network shrank and condensed around the nucleus in mt‐Ty 5′ gapmer treated cells (Figure 5A). To further analyse mitochondrial network changes in gapmer‐treated cells, the MiNA plugin^18^ was used with ImageJ to process the mitochondrial staining images and determine their framework structure. Individual mt with a distinct rod/puncta appearance were decreased significantly (*p* < 0.0001) from 468 counts/cell on average in Ctrl gapmer treated cells to 221 counts/cell on average in mt‐Ty gapmer treated cells (Figure S3A). Mitochondrial network number was similarly decreased from 42 counts/cell in Ctrl cells to 22 counts/cell in mt‐Ty gapmer treated cells (Figure S3B; *p* < 0.0001). There was no significant difference in mitochondrial mean branch length between the two groups (Figure S3C). Mitochondrial volume, defined as the total signal from the mitochondrial footprint, was decreased significantly (*p* < 0.0001) from 16,273 counts/cell in Ctrl cells to 7284 counts/cell in mt‐Ty gapmer treated cells (Figure S3D).

Since quantification of mitochondrial parameters by MiNA suggested that 5′ gapmer treatment induced obvious disruptions in mt (Figure S3), we decided to further examine mitochondrial number and morphology by TEM. We found that mt in mt‐Ty 5′ gapmer treated cells were much smaller than that in Ctrl gapmer treated cells (Figure 5C). Quantification of mitochondrial surface area showed that the average size of mt in mt‐Ty 5′ gapmer treated C2C12 myoblasts was 0.18 μm^2^, significantly lower than the 0.28 μm^2^ value determined for control cells (Figure 5D; *p* < 0.0001). We also determined the mt number in a specific area (per 1.1 μm^2^ image field) to identify the mt density; we found that there were 3.2 and 6.4 mt per 1.1 μm^2^ on average in Ctrl and mt‐Ty 5′ gapmer treated C2C12 myoblasts, respectively (Figure 5E). To further examine whether the mitochondrial fragmentation observed was a result of mt‐Ty 5' tiRNA inhibition, we performed GSEA analysis on genes related to mitochondrial organization. We found that genes associated with mitochondrial organization showed no significant change in gene expression in mt‐Ty 5′ gapmer treated cells (Figure 5F); these data suggest that mitochondrial dysregulation is a secondary outcome from mt‐Ty 5' tiRNA inhibition. Given that mt play an important role in the process of cell apoptosis,^31^, ^32^ evidence of mitochondrial fragmentation in mt‐Ty gapmer treated cells further implicates mt‐Ty 5' tiRNAs in the regulation of apoptosis in skeletal muscle.

### 3.6 Ectopic expression of mt‐Ty 5'tiRNAs affects expression of genes related to muscle development

**FIGURE 6:**
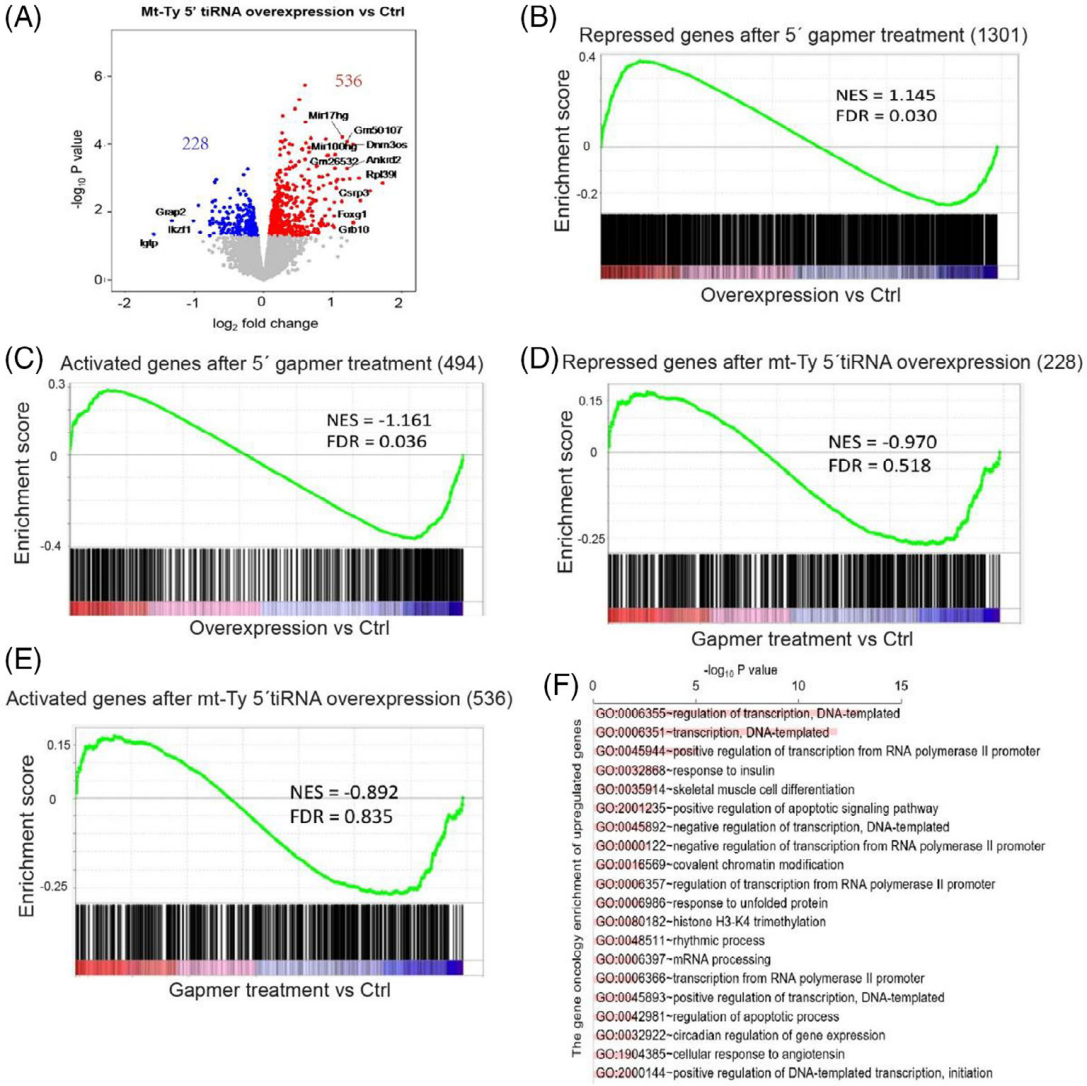
*Ectopically expressed mt‐Ty 5'tiRNAs in proliferating C2C12 cells promote expression of genes involved in muscle differentiation*. (A) The volcano plot of gene expression changes in Ctrl RNA versus mt‐Ty 5'tiRNA treated C2C12 myoblasts. Reduced genes (Fold Change <−1.5, pAdj <0.1) are coloured blue; activated genes (Fold Change >1.5, pAdj <0.1) are coloured red. (B) GO terms of activated (red) gene profile in C2C12 myoblasts with overexpression of mt‐Ty 5'tiRNAs (pAdj). (C) GSEA analysis of genes differentially expressed in mt‐Ty 5'tiRNA treated C2C12 myoblasts to compare their enrichment with repressed genes from 5′ gapmer treated cells. (D) GSEA analysis of genes differentially expressed in mt‐Ty 5'tiRNA treated C2C12 myoblasts to test their enrichment with activated genes from 5′ gapmer treated cells. (E) GSEA analysis of genes differentially expressed in 5′ gapmer treated C2C12 myoblasts to compare their enrichment with repressed genes from mt‐Ty 5'tiRNA treated cells. (F) GSEA analysis of genes differentially expressed in 5′ gapmer treated C2C12 myoblasts to test their enrichment with activated genes from mt‐Ty 5'tiRNA treated cells.

Next, we ectopically overexpressed mt‐Ty 5'tiRNA in C2C12 myoblasts. Unexpectedly, we did not observe any obvious changes in cell morphology or cell number, and there was no significant change in cell proliferation by quantification of pH 3‐positive cells (determined in mt‐Ty 5'tiRNA transfected cells compared to control RNA, data not shown). Thus, we performed RNA‐Seq analysis for RNAs extracted from C2C12 myoblasts transfected with Ctrl RNAs or synthetic mt‐Ty 5'tiRNAs (~50 million reads per sample, >99% high quality reads, and ~ 90% total mapping rate) (Figure S1). Consistent with the lack of obvious changes in cell morphology and number, we did not observe a significant increase in differentially expressed genes and any gene expression changes were low (FC < =2). If we repeat our analyses with a slightly modified *p‐*value (*p* < =0.05 and FC < =0.58), we detect 764 differentially expressed genes. In contrast to the results of our analyses of mt‐Ty 5'tiRNA gapmer treated cells, overexpression of mt‐Ty 5'tiRNA led to a greater number of upregulated genes than downregulated genes (Figure 6A). Compared with controls, 536 genes were upregulated, while 228 genes were downregulated in samples where mt‐Ty 5'tiRNA was overexpressed (Figure 6A). Two of the genes that were enriched after overexpression of mt‐Ty 5'tiRNA were Egr1 and Prl2c2. Egr1 (Early growth response 1) gene is a transcription factor which is required for differentiation of many cell types^33^, ^34^, ^35^; Prl2c2 enhances cell growth and plays an important role in embryonic development.^36^, ^37^ We validated the results by RT‐PCR and demonstrated that both genes were expressed at higher levels in mt‐Ty 5'tiRNA overexpression samples (*p* = 0.09 for Egr1, *p* = 0.02 for prl2c2) (Figure S4).

We also compared the expression pattern of genes between the 5′ gapmer and mt‐Ty 5'tiRNA treated cells and found they showed an inverse correlation. The repressed genes in mt‐Ty 5'tiRNA depleted C2C12 myoblasts were significantly upregulated in mt‐Ty 5'tiRNA overexpressing cells (Figure 6B, NES = 1.145. FDR = 0.03). Consistent with this observation, genes activated in mt‐Ty 5'tiRNA depleted cells were significantly downregulated in mt‐Ty 5'tiRNA overexpressing cells (Figure 6C, NES = −1.161, FDR = 0.036; Figure 5D). These results demonstrate that overexpression or inhibition of mt‐Ty 5'tiRNA modulates the skeletal muscle cell proliferation and differentiation programs. In contrast, neither a positive, nor a negative correlation was detected when genes with decreased or increased expression in both mt‐Ty 5'tiRNA overexpressing cells and mt‐Ty 5'tiRNA gapmer treated cells were compared (Figure 6D, E), NES = −0.970, FDR = 0.518 and NES = −0.892, FDR = 0.835, respectively. In mt‐Ty 5'tiRNA overexpressing cells, GO‐enriched terms for genes related to transcription were significantly increased. We also noticed that the ‘skeletal muscle cell differentiation’ category of genes was also upregulated, supporting a role for mt‐Ty 5'tiRNAs in muscle differentiation (Figure 6F). Collectively, overexpression of mt‐Ty 5'tiRNA promoted the expression of a small set of genes which are critical for the regulation of cell growth and development. This corresponds well with our earlier observation that depletion of mt‐Ty 5'tiRNAs led to inhibition of cell proliferation and differentiation. Therefore, we propose that these data support a critical role for mt‐Ty 5'tiRNAs in skeletal muscle function.

## 4 DISCUSSION

Conditions, such as oxidative stress, nutritional deficiency, and hypoxia, have been shown to induce the expression of tiRNAs.^5^, ^38^, ^39^ An increasing number of studies have also implicated specific tiRNAs in the promotion of cell proliferation^8^ and cancer cell migration,^40^ and the inhibition of stem cell pluripotency.^7^ However, very few studies have investigated the role of tiRNAs in muscle development. We initially identified several tsRNAs including mt‐Ty, mt‐Tc, mt‐Tv, and Val/His/Asp/Gln/Glu/Gly/Lys 5'tiRNAs that are highly enriched in 2mo skeletal muscle; we decided to focus on mt‐Ty 5'tiRNA since it is the most abundant of these molecules. We determined that mt‐Ty 5'tiRNAs were specifically expressed in skeletal muscle and heart; they also increased in abundance with age. This pattern of expression indicated that mt‐Ty 5'tiRNAs could play important roles in muscle development. Therefore, we employed the muscle‐derived C2C12 cell line to further investigate the functions of mt‐Ty 5'tiRNA in muscle development.

To characterize the function of mt‐Ty 5'tiRNA in muscle, we inhibited mt‐Ty 5'tiRNAs in C2C12 myoblasts using gapmers. Depletion of mt‐Ty 5'tiRNAs led to a significant decrease in cell number, which was subsequently determined to be a result of decreased cell proliferation and induction of apoptosis. In addition, knockdown of mt‐Ty 5'tiRNAs in C2C12 myoblasts prevented myotube formation in these cells even after 3 days of treatment to induce differentiation. Our RNA‐Seq data also demonstrated that depletion of mt‐Ty 5'tiRNAs altered expression of genes that are highly involved in proliferation, differentiation, and apoptosis. Since these pathways are recruited during the induction of myoblasts into myotubes, these data provide insights into the molecular mechanisms by which mt‐Ty 5'tiRNA knockdown is able to prevent this differentiation process.

Analysis of mitochondrial morphology revealed that their structure was disrupted in mt‐Ty 5'tiRNA depleted cells; specifically, both mitochondrial size and number were decreased. Mitochondrial disruption can have severe adverse effects on cellular health since they are especially important for normal cardiac and skeletal myoblast function.^41^, ^42^, ^43^, ^44^, ^45^ Not surprisingly, mitochondrial dysfunction often inhibits cell proliferation and causes cell death.^46^, ^47^ Therefore, we deemed it important to investigate whether the change in mitochondria is a direct result of mt‐Ty 5'tiRNA depletion. Our RNA‐Seq analyses showed that mitochondrial organization and apoptosis gene pathways were not significantly altered in mt‐Ty 5'tiRNA depleted cells; these data are consistent with a model in which the observed mitochondrial fragmentation resulted from apoptosis rather than causing it.

In this study, we also ectopically expressed mt‐Ty 5'tiRNAs in C2C12 cells to determine if any changes would be induced. Although there was no obvious phenotype upon visual inspection, we determined that 536 genes were activated and 228 genes were repressed after mt‐Ty 5'tiRNA overexpression; these data were in contrast to those determined following mt‐Ty 5'tiRNA depletion, in which there were 1301 repressed genes, significantly more than the number of activated genes. To date, the primary molecular mechanism associated with mt‐Ty 5'tiRNA function has been reported to be translational regulation.^3^, ^6^ It is intriguing that the activated genes we detected upon mt‐Ty 5'tiRNA overexpression (Egr1, Snhg17, Prl2c2, and Errfi1) are involved in transcriptional regulation and are also required for cell proliferation and development.^33^, ^34^, ^35^, ^36^, ^37^, ^48^, ^49^ The data we report here support a role for mt‐Ty 5'tiRNAs as important regulators of gene expression that are enriched in muscle during development. We further propose that they function in muscle through the regulation of cell proliferation and differentiation pathways.

### AUTHOR CONTRIBUTIONS STATEMENT

Conceptualization, Jun Cao, Vivek Advani, Yao Wei Lu, Andrea P. Malizia, Gurinder Bir Singh, Zhan‐Peng Huang, Jianming Liu, and Da‐Zhi Wang; methodology, Jun Cao, Xin Wang, Vivek Advani, Yao Wei Lu, Andrea P. Malizia, Gurinder Bir Singh, Zhan‐Peng Huang, Jianming Liu, Chunbo Wang, and Edilamar M. Oliveira; software, Jun Cao, Xin Wang, Vivek Advani, Yao Wei Lu, Andrea P. Malizia, Gurinder Bir Singh, Zhan‐Peng Huang, Jianming Liu, Chunbo Wang, Kaifu Chen, and Da‐Zhi Wang; validation, Jun Cao, Xin Wang, Vivek Advani, Yao Wei Lu, Andrea P. Malizia, Gurinder Bir Singh, Zhan‐Peng Huang, Kaifu Chen, and Da‐Zhi Wang; formal analysis, Vivek Advani, Yao Wei Lu, Kaifu Chen, and Da‐Zhi Wang; investigation, Jun Cao, Xin Wang, Vivek Advani, Yao Wei Lu, Andrea P. Malizia, Gurinder Bir Singh, Zhan‐Peng Huang, Jianming Liu, Chunbo Wang, Edilamar M. Oliveira., Kaifu Chen, and Da‐Zhi Wang; resources, Kaifu Chen and Da‐Zhi Wang; data curation, Jun Cao, Zhan‐Peng Huang, Kaifu Chen, and Da‐Zhi Wang; writing (original draft preparation), Jun Cao, Vivek Advani, Yao Wei Lu, Andrea P. Malizia, and Da‐Zhi Wang; writing (review and editing), Jun Cao, John D. Mably, Da‐Zhi Wang; visualization, Jun Cao, Xin Wang, Vivek Advani, Yao Wei Lu, Andrea P. Malizia, Gurinder Bir Singh, Zhan‐Peng Huang, Jianming Liu, and Chunbo Wang; supervision, Da‐Zhi Wang; project administration, Kaifu Chen and Da‐Zhi Wang; funding acquisition, Da‐Zhi Wang All authors have read and agreed to the published version of the manuscript.

## FUNDING INFORMATION

This research was funded by the National Institutes of Health (NIH), grant numbers NIH HL149401 and NIH HL138757. Jun Cao was supported by a postdoctoral fellowship from the American Heart Association (18POST3399018) and National Natural Science Foundation of China (82200328). The contents of the manuscript are solely the responsibility of the authors and do not necessarily represent the official views of the NHLBI or NIH. Edilamar M. Oliveira is supported by an award from FAPESP (Brazil), grant number 2022/00531‐5.

## CONFLICT OF INTEREST STATEMENT

The authors declare no conflicts of interest.

## INSTITUTIONAL REVIEW BOARD STATEMENT

This work was reviewed and approved by the Boston Children's Hospital Institutional Animal Care and Use Committee (IACUC), protocol number 18‐08‐3759R.

## INFORMED CONSENT STATEMENT

Not applicable.

## Supporting information


**Data S1.** Supporting Information.

## Data Availability

We have based this plan on those successfully used with our NIH awards. We are committed to the open and timely dissemination of our research findings and recognize that promising new methods, technologies, data, software programs, and scientific insights may arise during this research. We are aware of and agree to abide by the principles for sharing research resources, as described by the National Institutes of Health (NIH) in the document entitled “Principles and Guidelines for Recipients of NIH Research Grants and Contracts on Obtaining and Disseminating Biomedical Research Programs.” The data generated in this grant will be presented at regional, national, or international conferences and published in a timely fashion. All final peer‐reviewed and non‐peer‐reviewed manuscripts that arise from this proposal will be submitted to the digital archive PubMed Central and, wherever possible, the data contained in these publications, as well as all other data generated by this project, will be deposited in other appropriate public repositories (e.g., Figshare).Data generated under this project will be administered in accordance with both the University of South Florida (USF) and NIH policies, including the NIH Data Sharing Policy and Implementation Guidance of March 5, 2003. We will adhere to the NIH Public Access Policy to ensure the timely release and sharing of final research data from NIH‐supported studies for use by other researchers. All published papers, abstracts, and proceedings will be available to the general public, including both the academic and industrial sectors. Should any intellectual property arise which requires a patent, we will ensure the technology (i.e., materials and data) is available to the research community in accordance with the NIH Best Practices for the Licensing of Genomic Inventions and Section 8.2.3, Sharing Research Resources, of the NIH Grants Policy Statement. In other words, there will be no restrictions or limits placed on the sharing of data generated from this project. Material transfers will be made with no more restrictive terms than in the Simple Letter Agreement (SLA) or the Uniform Biological Materials Transfer Agreement (UBMTA) and without ‘reach‐through’ requirements.My laboratory has shown a commitment to sharing by providing cell lines, antibodies, plasmid constructs, and mice over the past 15 years. Any unique reagents that might be developed as a result of the research project will be made readily available to the scientific community. We will provide relevant protocols and published genetic and phenotypic data upon request. We will adhere to the NIH Grant Policy on sharing of unique research resources as outlined in the publication entitled “Sharing of Biomedical Research Resources Principles and Guidelines for Recipients of NIH Grants and Contracts”. “Other Research Resources” generated with funds from this grant may include DNA constructs and sequence data. These resources would be freely distributed upon request for non‐commercial research. Any sequence data from bulk and single cell RNA‐sequencing experiments will be deposited in a public database such as GEO upon publication.We assume responsibility for distributing newly generated model organisms and we will fulfill these requests in a timely fashion. Following the characterization and peer‐reviewed publication of mouse strains, they will be freely distributed to investigators at academic institutions wanting mice for non‐commercial research. Recipient investigators will provide written assurance and evidence that the animals will be used solely in accord with appropriate IACAC review, that the recipient will not further distribute animals without our consent, and that animals will not be used for commercial purposes. To facilitate sharing and distribution of the transgenic mice and associated resources developed under this grant, mice will be maintained in a pathogen‐free facility.
