# Supplementary material for: mt‐Ty 5'tiRNA regulates skeletal muscle cell proliferation and differentiation
Source: Cell Prolif. 2023 Feb 8;56(8):e13416. doi: 10.1111/cpr.13416 (PMC10392060; doi:10.1111/cpr.13416)
Supplement: Supplementary file 1 — Data S1. Supporting Information. [file CPR-56-e13416-s001.docx]

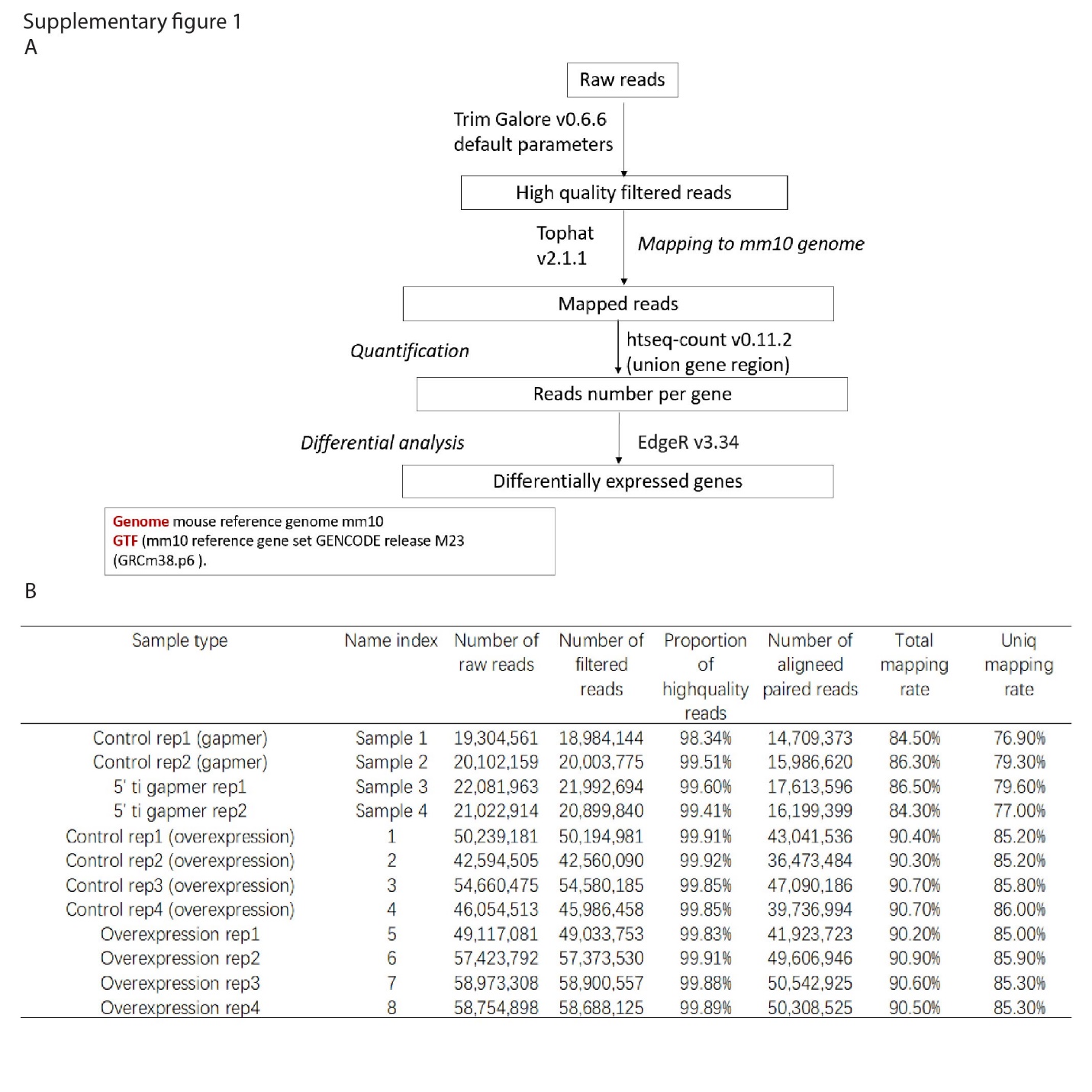


**Supplementary Figure 1. RNA-Seq workflow and sample alignment information.** A) Diagram of RNA-Seq process. B) RNA-Seq sample mapping rate.


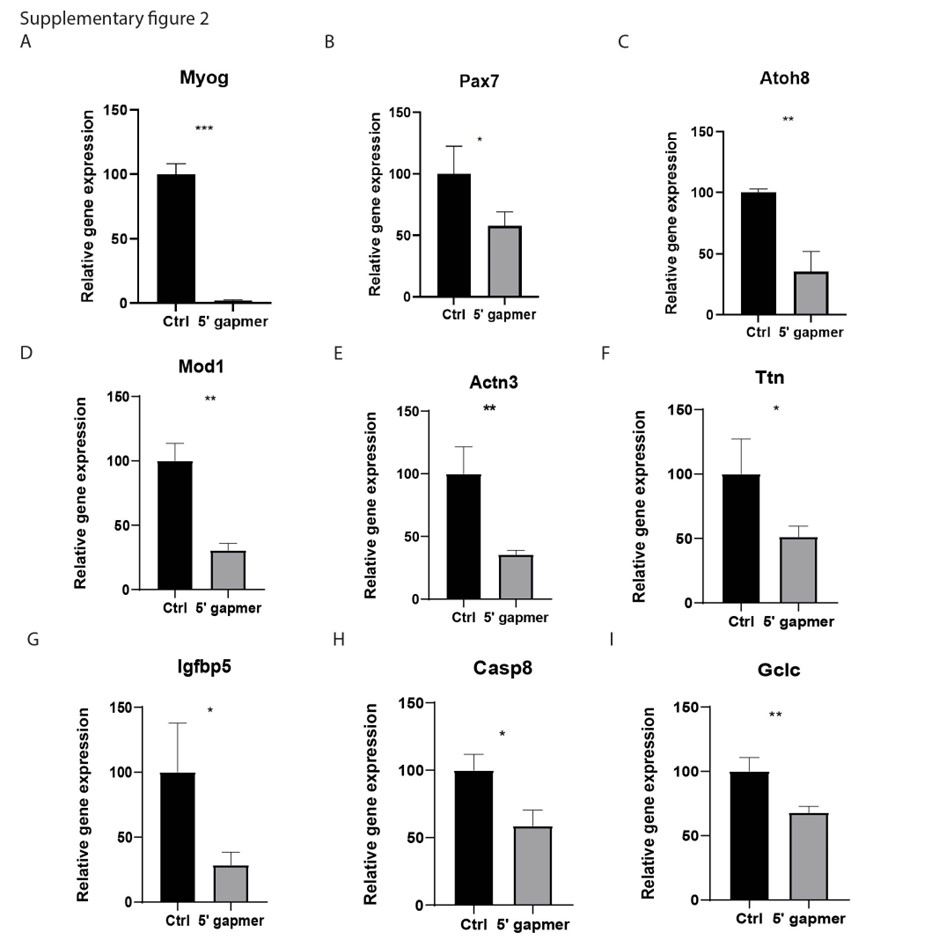


**Supplementary Figure 2. Relative expression of proliferation, differentiation and apoptosis related genes in Ctrl and 5´gapmer treated cells.** Gene expression level of A) Myog, B) Pax7, C) Atoh8, D) Mod1, E) Actn3, F) Ttn, G) Igfbp5, H) Casp8, I) Gclc in Ctrl and 5´gapmer treated C2C12 myoblasts. Data represent means ± SD. Statistical significance was calculated using t-test to compare two different groups in three independent experiments (n=3). *p<0.05, **p<0.01, ***p<0.001.


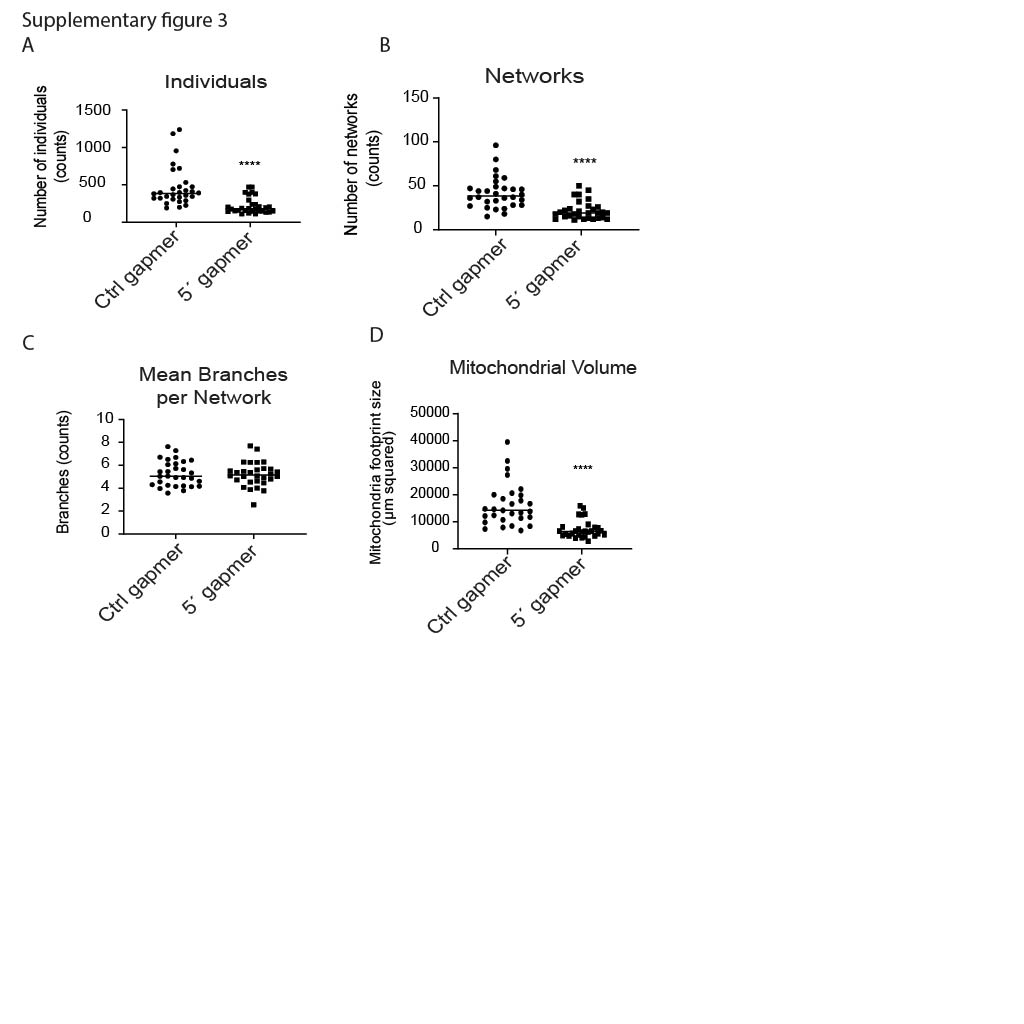


**Supplementary Figure 3. Mitochondria analysis from Tomm20 immunofluorescent staining.** A) Quantification of mitochondrial individuals (rod/punctate shapes of mitochondrial structures which do not contain a junction pixel) in Ctrl versus 5´ gapmer treated C2C12 myoblasts. Data represent means ± SD. Statistical significance was calculated using t-test to compare two different groups in three independent experiments (n=3). ****p<0.0001. (B) Quantification of mitochondrial networks (mitochondrial structures contain at least 1 junction pixel and are composed of more than one branch) in Ctrl versus 5´ gapmer treated C2C12 myoblasts. Data represent means ± SD. Statistical significance was calculated using t-test to compare two different groups in three independent experiments (n=3). ****p<0.0001. (C) Quantification of mitochondrial mean branch length (the mean length of all the lines used to represent the mitochondrial structures) based on MiNA analysis in Ctrl versus 5´ gapmer treated C2C12 myoblasts. Data represent means ± SD. Statistical significance was calculated using t-test to compare two different groups in three independent experiments (n=3). (D) Quantification of mitochondrial volumes (mitochondrial footprint volumes depicting all the mitochondrial immunofluorescent signal after being separated from the background) in Ctrl versus 5´ gapmer treated C2C12 myoblasts. Data represent means ± SD. Statistical significance was calculated using t-test to compare two different groups in three independent experiments (n=3). ****p<0.0001.


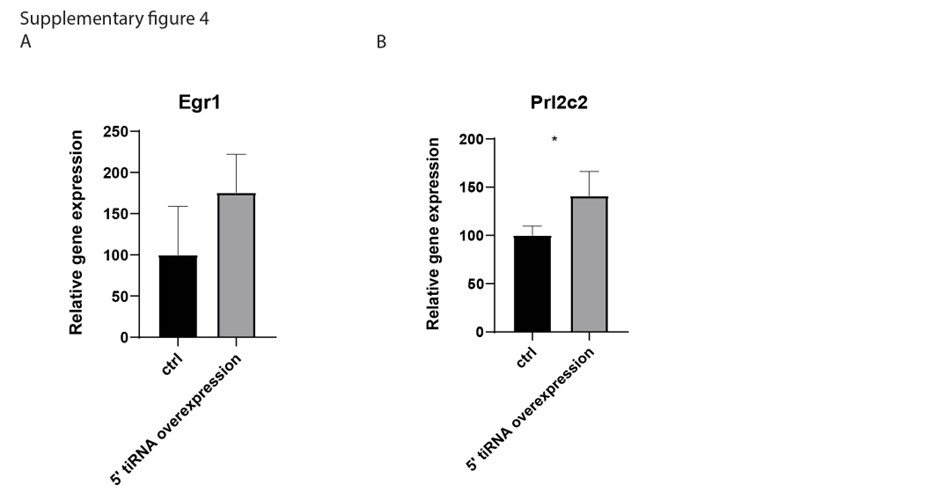


**Supplementary Figure 4. Relative expression of Egr1 and Prl2c2 in Ctrl and 5´tiRNA overexpressed cells.** Gene expression level of A) Egr1(p=0.09), B) Prl2c2 (*p<0.05) in Ctrl and 5´tiRNA overexpressed cells. Data represent means ± SD. Statistical significance was calculated using t-test to compare two different groups in three independent experiments (n=3).

| **Near-infrared fluorescent northern blot IR-dye conjugated oligos** | | | |
| --- | --- | --- | --- |
| **Target gene** | **oligo sequence** | | |
| U6 | 5´-GCAGGGGCCATGCTAATCTTCTCTGTATCGT/3AzideN/-3´ | | |
| Mt-Ty 5´ tiRNA | 5´-TCTAATGCTTACTCAGCCATTTTACC/3AzideN/-3´ | | |
| **5´ primer for DNA plasmid sequencing** | | | |
| 5´-GACCACGCGTATCGGGCACCACGTATGCTATCGATCGTGAGATGGG-3´ | | | |
| **Gapmers** | | | |
| **Gapmer name** | **Target region** | **Oligo sequence** | |
| Ctrl gapmer | NA | 5´-+C*+G*+T*+A*C*G*C*G*G*A*A*T*A*C*+T*+T*+C*+G-3´ | |
| Mt-Ty gapmer | 5´-GGTAAAATGGCTGAGTAAGC-3´ | 5´-+G*+C*+T*+T*A*C*T*C*A*G*C*C*A*T*T*T*+T*+A*+C*+C -3´ | |
| **RNA mimics** | | | |
| **RNA mimic name** | **Oligo sequence** | | |
| Ctrl RNA | /5Phos/rGrCrGrArCrUrArUrArCrGrCrGrCrArArUrArUrG | | |
| mt-Ty 5´tiRNA | /5Phos/rGrGrUrArArArArUrGrGrCrUrGrArGrUrArArGrCrArUrUrArGrArCrUrGrUrA | | |
| **qPCR primers** | | | |
| Target gene | Forward primer | | Reverse primer |
| Gapdh | GCACAGTCAAGGCCGAGAAT | | GCCTTCTCCATGGTGGTGAA |
| HprT | CCTCCTCAGACCGCTTTTT | | AACCTGGTTCATCATCGCTAA |
| Myog | CCATCCAGTACATTGAGCGCCT | | CTGTGGGAGTTGCATTCACTGG |
| Pax7 | GGCACAGAGGACCAAGCTC | | GCACGCCGGTTACTGAAC |
| Atoh8 | CGGGGGAAAGTTCCTACTCGTC | | CGGAAGAATCCGGGTGGTTATT |
| Mod1 | AGCACTACAGTGGCGACTCA | | GGCCGCTGTAATCCATCA |
| Ttn | ATTGAAGCCCACTTTGATGC | | ACTGGGGAGGGAGAGTGTCT |
| Casp8 | TGCTTGGACTACATCCCACA | | GAAATCTGGGCATTGTCTGG |
| Gclc | CGGAGGAACGATGTCTGAGT | | CTCTGGGTTGGGTCTGTGTT |
| Actn3 | TGTCCCATGGCTAGAGAACC | | GGTCTGCAGCGTGTTGAAGT |
| Igfbp5 | TCCCTGCACCTGAGATGAGA | | GCACTGAAAGTCCCCATCCA |
| Egr1 | GAGGAGATGATGCTGCTGAG | | TGCTGCTGCTGCTATTACC |
| Prl2c2 | TGAGGAATGGTCGTTGCTTT | | TCTCATGGGGCTTTTGTCTC |

**Supplementary Table 1. Oligomer and primer sequences.**
